# 

**Published:** 1896-02

**Authors:** 


### MICHIGAN STATE VETERINARY ASSOCIATION.

THE Michigan State Veterinary Association will hold its fourteenth annual meeting at Lansing, on the 4th and 5th of the present month. On the 5th there will be practical demonstrations of laryngotomy, ovariotomy, castration of cryptorchids, and other minor operations. Papers will be presented on the following subjects:

"Some Observations on Conception and Pregnancy in the Mare," by J. A. Dill, Ann Arbor; "The Veterinary Surgeon," by C. M. Higginson, Jackson; "Foreign Matter in Alimentary Canal," by Judson Black, Richmond; "Extemporaneous Talk on Tuberculosis and Other Things," by E. A. A. Grange, Lansing; and a paper by Dr. George C. Moody-Mason. Such a programme should enlist the interest and attendance of every progressive veterinarian in the State.

### PERSONAL.

Veterinarian Tremaine, of Bridgeton, N. J., takes an active interest in secret societies. He was recently elected to one of the highest offices in the Imperial Order of Red Men.

Jno. Doris, Jr., D.V.S., Veterinary Inspector, Bureau of Animal Industry, U. S. Department of Agriculture, with station at Pittsburg, Pa., has tendered his resignation, which was accepted December 31, 1895. He has a well-equipped and modern veterinary hospital, and will hereafter devote his entire attention to private practice.

George Jobson, V.S., D.V.S., who was for three years Resident Surgeon and Professor of Anatomy at the National Veterinary College and Hospital in Washington, D. C., has located in private practice in Oil City, Pa., and is veterinarian to the Miller & Sibley stock farm at Franklin, Pa.

J. Stewart Lacock, V.M.D., of Allegheny, Pa., has opened a nice office, centrally located, in that city, and has secured the veterinary practice of the Department of Public Safety, which includes the fire, electric, public highways, police, and water bureaus.

Dr. W. J. Reagan, of Philadelphia, graduate of the veterinary department of the University of Pennsylvania, has recently passed a very successful examination for inspector in the Bureau of Animal Industry, and has been assigned to duty in New York.

Dr. R. R. Dinwiddie, of Arkansas, has been spending a season in St. Louis, Mo.

Henry J. Hancock, M.R.C.V.S., and James A. Waugh, V.S., are contributors to the first number of the *Humane Advocate*, just established by the Western Pennsylvania Humane Society of Pittsburg, Pa.

C. D. Smead, V.S., of Logan, N. Y., conducts the veterinary column of the *National Stockman and Farmer*.

Veterinarian E. R. Voorhees, of New Jersey, will shortly accept the post of veterinarian at the New York Agricultural Experiment Station, Geneva, N. Y., vice Dr. Peter Collier, resigned.

Dr. Donald McIntosh, Professor of Veterinary Science, University of Illinois, addressed the Illinois Swine-breeders' Association on January 8th, on "Health of Hogs."[1]

Dr. A. F. Peters recently addressed the Nebraska Dairymen's Association on "Anthrax." He recommended preventive vaccination in communities where the disease was widespread.

Dr. R. A. Archibald, of Sacramento, Cal., has removed to San Francisco.

Dr. James Johnstone, of St. Joseph, Mo., has recently been appointed to a position as assistant inspector, and been assigned to a place at the Philadelphia abattoir.

Dr. W. J. Hinman, of Winnipeg, Manitoba, fills the rôle of city veterinarian for that municipality. In connection with important city ordinances governing the milk-supply the position has become a very important one.

Dr. P. H. Seltzer, of Lebanon, recently addressed the Farmers' Institute of York County, which was held under the direction of the State Board of Agriculture.

Drs. J. Miller, of Ottumwa, and H. G. Moore, of Ames, Ia., have been appointed to assistant inspectorship in the Bureau of Animal Industry.

It is gratifying to know that the many favorable laws in Manitoba governing matters pertaining to the true growth and protection of veterinarians have been strongly aided by the presence, as members, in Manitoba's Parliament, of Veterinarians J. G. Rutherford, Dr. H. McFadden, and D. McNought.

[1] Will appear in March issue of JOURNAL.

Dr. O'Shea, of New York City, has gone to Albany to appear before the Committee on Public Health to defeat a bill introduced by Representative Cole, of Syracuse, extending the time of registration in New York State.

We learn that Professor Liautard intends to examine his students in anatomy the latter part of January, after which he sails for France for an indefinite stay. Prof. R. R. Bell is to take his place.

Mr. Roberge and Dr. Ferster will deliver a series of lectures at the Toronto Veterinary College, commencing January 22d, on "Balancing a Horse's Foot."

Thomas Giffen, M.R.C.V.S., of New York City, has adopted for his professional calls the latest design of rubber-tired hansom cab.

Dr. O'Dea, Casanovia, N. Y., was a New York visitor recently, calling at the various colleges and upon a number of practitioners.

Dr. W. L. Baker, of Cortlandt, N. Y., wants to sell his practice. On account of the ill-health of his wife he is obliged to leave for a milder climate.

Dr. Robert Richards, New York City, was lately elected Master of the Eastern Star Masonic Lodge, vice Dr. A. K. Robertson, of Brooklyn. There are between fifteen and twenty veterinarians members of this lodge, and it has been dubbed "The horse-doctors' lodge." Dr. Richards recently met with an accident, and was thrown from his wagon and considerably bruised.

